# Unraveling the Mystery of Hemoglobin in Hypoxia-Accelerated Neurodegenerative Diseases

**DOI:** 10.3390/biom15091221

**Published:** 2025-08-25

**Authors:** Zhengming Tian, Feiyang Jin, Zhuowen Geng, Zirui Xu, Qianqian Shao, Guiyou Liu, Xunming Ji, Jia Liu

**Affiliations:** 1 Beijing Institute of Brain Disorders, Laboratory of Brain Disorders, Hypoxia Conditioning Translational Laboratory of Clinical Medicine, Ministry of Science and Technology, Collaborative Innovation Center for Brain Disorders, Capital Medical University, Beijing 100054, China; tianzm@mail.ccmu.edu.cn (Z.T.);; 2 Chinese Institutes for Medical Research, Beijing 100069, China; 3 Department of Neurosurgery, Xuanwu Hospital, Capital Medical University, Beijing 100054, China; 4 School of Medicine, University of Leeds, Leeds LS2 9JT, UK

**Keywords:** aging, hypoxia, acute/chronic neurodegenerative diseases, cerebral hemoglobin, circulating hemoglobin, non-oxygen-binding functionality

## Abstract

Hypoxic stress is increasingly recognized as a convergent pathological factor in various age-related neurodegenerative diseases (NDDs), encompassing both acute events such as stroke and traumatic brain injury (TBI), and chronic disorders including Parkinson’s disease (PD), Alzheimer’s disease (AD), and amyotrophic lateral sclerosis (ALS). Recent studies have revealed that hemoglobin (Hb), beyond its classical oxygen-transport function, exhibits unexpected expression and functional relevance within the central nervous system. Notably, both cerebral and circulating Hb appear to be dysregulated under hypoxic and aging conditions, potentially influencing disease onset and progression of these diseases. However, Hb’s impact on neurodegeneration appears to be context-dependent: in acute NDDs, it may exert neuroprotective effects by stabilizing mitochondrial and iron homeostasis, whereas in chronic NDDs, aberrant Hb accumulation may contribute to toxic protein aggregation and neuronal dysfunction. This review provides an integrative overview of the emerging roles of Hb in hypoxia-related NDDs, highlighting both shared and distinct mechanisms across acute and chronic conditions. We further discuss potential therapeutic implications of targeting Hb-related pathways in NDDs and identify key gaps for future investigation.

## 1. Introduction

The 2022 United Nations World Population Prospects report projected that the global population aged 65 years and older will increase from 10% in 2022 to 16% by 2050 [1]. As the elderly population increases, the economic burden of age-related diseases is expected to rise accordingly [2]. Aging leads to a progressive decline in tissue and organ function, and it is now well established that aging is the primary risk factor for many neurodegenerative diseases (NDDs), though there is a notable lack of effective treatments [3].

NDDs are a heterogeneous group of disorders of the nervous system that are primarily characterized by progressive dysfunction and the loss of neurons and axons within the central nervous system. Globally, these diseases have become a leading cause of disability and premature death among the elderly [4,5,6]. Recent studies suggest that in addition to chronic NDDs (such as Parkinson’s disease, Alzheimer’s disease, and amyotrophic lateral sclerosis), acute brain diseases, such as stroke and traumatic brain injury, can also be classified as acute NDDs [7,8]. However, the distinction between acute and chronic NDDs remains ambiguous, and a systematic comparison of the commonalities and differences between the core mechanisms of these diseases has yet to be undertaken.

Hypoxia is a major factor in brain aging, and previous studies have shown that hypoxia in the brain intensifies with age [9,10]. Hypoxia is a hallmark pathological feature in both acute and chronic NDDs, although its precise role remains to be fully elucidated [11,12,13].

Hemoglobin (Hb), as the primary protein responsible for oxygen transport and gas exchange in the body [14], has been unexpectedly identified in various non-erythroid cells, including neurons and glial cells in the brain. The function of Hb is not limited to gas exchange and transport; it participates in other significant physiological processes, such as maintaining mitochondrial function and mediating the clearance of toxic proteins [15,16,17,18,19,20]. These findings suggest that Hb may play a crucial role in NDDs related to hypoxia, thus warranting further investigation. For clarity, we define circulating hemoglobin as erythrocyte-derived Hb involved in systemic oxygen transport, whereas cerebral hemoglobin refers to endogenously expressed Hb within the neural and glial cells of the central nervous system. These forms are distinct in their cellular origin and localization, and may exert divergent functional roles in the context of neurodegeneration.

In this review, we define and discuss uniformity and heterogeneity in the onset age and primary pathology of both acute and chronic NDDs, and confirmed aging as the predominant shared risk factor with hypoxia identified as a critical common pathology. Based on the discovery of abnormal Hb expression and its non-canonical functionality in the central nervous system (CNS), we emphasize the importance of Hb in the CNS. We also describe the key mechanisms regulating Hb expression and functionality under hypoxic stress and highlight the significant role of Hb in neurodegenerative diseases, such as stroke, traumatic brain injury, Parkinson’s disease, Alzheimer’s disease, and amyotrophic lateral sclerosis, in which hypoxia is a key pathological feature. In summary, we offer a novel perspective for understanding these diseases and for developing innovative therapeutic strategies.

## 2. Defining the Role of Hypoxia in Acute and Chronic NDDs

NDDs encompass a range of chronic conditions, including Alzheimer’s disease (AD), Parkinson’s disease (PD), and amyotrophic lateral sclerosis (ALS), as well as acute diseases, such as stroke and traumatic brain injury (TBI). However, the definition and differentiation between acute and chronic NDDs have yet to be fully addressed. A systematic comparison of core mechanisms remains lacking, which limits the advancement of pathological research. In this review, we provide an epidemiological analysis and summarize the key pathological features of both acute and chronic NDDs, identifying aging as a primary risk factor and confirming hypoxia as a shared pathological hallmark. Furthermore, we explore the similarities and differences in hypoxia-regulated mechanisms across different NDDs, providing new perspectives for understanding the pathogenesis of disease and developing targeted therapeutic strategies.

### 2.1. Aging as the Primary Risk Factor for Both Acute and Chronic NDDs

NDDs are a diverse group of neurological disorders affecting millions worldwide. These diseases lead to the progressive dysfunction and loss of neurons and axons in the central nervous system, culminating in the breakdown of neural networks and impairment of memory, cognition, behavior, sensation, or motor function. NDDs are a leading cause of disability and premature death among the elderly [4,5,6]. Common NDDs, such as Alzheimer’s disease (AD), Parkinson’s disease (PD), and amyotrophic lateral sclerosis (ALS), are closely associated with aging and can be considered as disease states that are induced by abnormal aging [21,22]. These diseases typically progress slowly, with symptoms often manifesting in later stages of life, classifying these as chronic NDDs [7]. On the other hand, diseases such as ischemic stroke, hemorrhagic stroke, and TBI, which are characterized by rapid and massive neuronal death due to brain ischemia or hemorrhage, have been classified as acute NDDs [8]. Although this classification is not yet universally adopted, it provides a useful framework for examining the shared and divergent features of neurodegenerative processes [23,24]. In this review, we compare epidemiological data and key pathologies of these diseases to highlight their similarities and differences, thus providing a multidimensional perspective on mechanisms and therapeutic interventions for NDDs.

### 2.2. Hypoxia as a Common Pathological Feature in Acute and Chronic NDDs

Aging is widely recognized as the primary risk factor for both acute and chronic NDDs. Current research has clearly identified brain hypoxia as a key driving factor in aging. As individuals age, the hypoxic conditions in the brain tissue progressively worsen [9,10]. Here, we systematically review the major pathological features of both acute and chronic NDDs, demonstrating brain hypoxia as both a common feature and a major contributor to these aging-related diseases (Table 1). For example, the primary pathological mechanism underlying ischemic stroke is the interruption of cerebral blood flow, leading to hypoxia and glucose deprivation. Similarly, brain hemorrhage leads to secondary hypoxia and tissue damage, thus contributing to the pathology of hemorrhagic stroke and TBI [25,26]. In the pathological progression of PD, AD, and ALS, hypoxia influences disease development via various mechanisms, including impairing neuronal energy metabolism, inducing neuroinflammation, and promoting abnormal protein aggregation [27,28,29]. Understanding the adaptive mechanisms of the body in response to hypoxia is essential if we are to identify the pathogenic mechanisms underlying these hypoxia-related NDDs and develop more effective therapeutic strategies.

Older adults, especially those aged ≥70 years, are disproportionately affected by acute NDDs such as stroke [30]. Similarly, chronic NDDs predominantly affect individuals aged 65 years and older (Table 1) [31,32,33,34,35]. These epidemiological studies suggest that aging is the critical risk factor that is common to both acute and chronic NDDs. This finding highlights the central role of aging in the pathogenesis of NDD, thus providing vital clues for understanding the pathological mechanisms underlying these diseases.

**Table 1:** Classification, definition, epidemiology, and main pathologies of acute/chronic neurodegenerative diseases.

Classification	Disease	Proportion of Age Groups Among Patients	Main Pathological Mechanism
Acute NDDs	Stroke	~10% prevalence in general population; ~90% among individuals aged ≥70 years [30].	Ischemic stroke: ischemia; glucose deprivation; hypoxia [36]. Hemorrhagic stroke: cerebral hemorrhage; ischemia and hypoxia secondary to cerebral hemorrhage [25].
	Traumatic brain injury	/	Primary brain injury caused by external force; secondary brain hemorrhage, ischemia, and hypoxia [26].
Chronic NDDs	Parkinson’s disease	~20% prevalence among individuals aged <70 years; ~80% among those aged ≥70 years [31,32].	Progressive loss of dopaminergic neurons; abnormal protein degradation system; abnormal aggregation of α-syn; mitochondrial dysfunction-induced hypoxia; genetic factors [27,37].
	Alzheimer’s disease	~26% prevalence among individuals aged <75 years; ~74% among those aged ≥75 years [33].	Abnormal accumulation of amyloid-β plaques and tau neurofibrillary tangles; cerebrovascular disease; mitochondrial dysfunction-induced hypoxia; genetic factors [38,39].
	Amyotrophic lateral sclerosis	Average age of onset ~65 years [35], peak prevalence at ~75 years [34].	Abnormal accumulation of TDP-43 protein; genetic factors [28,29].

## 3. Hb: An Overlooked Research Target in Hypoxia–Aging Diseases

Hb is widely recognized for its role in oxygen binding and transport in red blood cells; its specific functions in mammalian red blood cells have been studied extensively [20]. However, an increasing body of evidence now supports the fact that Hb is more widely expressed than previously thought, with its presence confirmed in non-erythroid tissues, including the brain and muscles [40]. Consequently, considering the functions of Hb in addition to gas transport and exchange could provide new insights into the pathology of Hb-related pathologies and the development of potential therapeutic interventions.

### 3.1. Unexpected Expression and Functionality of Non-Erythroid Hemoglobin

Hb, the most abundant oxygen-binding protein, is primarily located in mature red blood cells and is referred to as circulating Hb [41,42]. Adult Hb is a heterotetramer composed of two alpha (Hbα) subunits encoded by *HBA1/2* and two beta (Hbβ) subunits encoded by *HBB*. The primary function of this form of Hb is to bind oxygen in the lungs and transport it through the large and small arteries and capillaries to peripheral tissues, where oxygen is exchanged for carbon dioxide, thus supporting aerobic respiration and the production of energy [43]. In addition, Hb plays a role in protecting cells from oxidative stress by reacting with hydrogen peroxide (H_2_O_2_) and nitric oxide (NO) [44,45].

Interestingly, Hb has also been identified in other non-erythroid tissues, including neurons, glial cells, endothelial cells, and retinal cells, suggesting potential roles for Hb beyond oxygen transport in diverse physiological and pathological contexts [15,16,17,18,19,20]. Of particular note is the expression of Hb in the CNS, where its presence in neurons, glial cells, and even mitochondria has been confirmed [20,46]. This form of Hb, expressed in the CNS, is referred to as cerebral Hb, and its functions extend beyond oxygen binding and transport. For example, neuronal Hb is known to be involved in oxygen storage and mitochondrial protection [47], while in glial cells, Hb plays a role in oxidative stress and iron metabolism [48,49]. Furthermore, endothelial Hb is known to be involved in the clearance of toxic NO [50]. Although increasing evidence highlights the cerebral expression of Hb, its precise mechanisms of action remain poorly characterized.

Research has shown that the α and β chains of Hb in the human brain co-localize with mitochondria, thus suggesting that cerebral Hb may be primarily localized within the mitochondria [51,52]. Both immunoelectron microscopy and mitochondrial fractionation studies have confirmed the interaction between the α and β chains of Hb and mitochondria, with their subcellular localization identified in the mitochondrial intermembrane space [53]. When the expression of neuronal Hb is upregulated, both cerebral oxygenation and mitochondrial activity increase significantly, thus suggesting a role for neuronal Hb in mitochondrial neuroprotection and oxygen homeostasis [47,54]. Furthermore, proteomic analysis of the proteins interacting with neuronal Hb has revealed a large number of mitochondrial proteins, further suggesting that neuronal Hb may be involved in mitochondrial energy production and transport [55]. Collectively, these findings indicate that cerebral Hb is predominantly localized in the mitochondria and may play a role in mitochondrial-mediated neuroprotection and oxygen regulation, although the precise molecular function of this form of Hb remains unclear.

Based on these discoveries, this review classifies Hb into circulating Hb and cerebral Hb to investigate differences in functionality and potential mechanisms in both acute and chronic NDDs from the perspective of hypoxic regulation. Our approach provides new insights into understanding NDDs and offers potential avenues for developing novel therapeutic interventions.

### 3.2. The Close Relationship Between Aging and Hb

**Figure 1:**
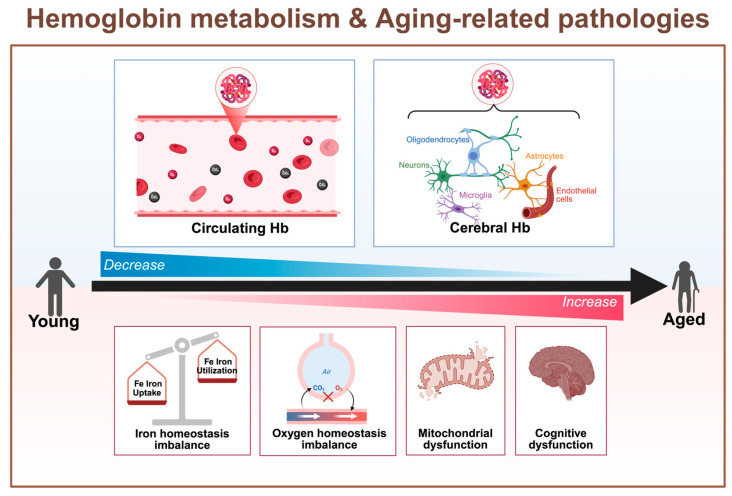
The levels of hemoglobin decrease with age. This protein plays a key role in oxygen metabolism, mitochondrial function, and cognitive dysfunction. With aging, both circulating and cerebral Hb levels gradually decline, leading to disruptions in iron homeostasis, oxygen homeostasis, mitochondrial dysfunction, and cognitive dysfunction.

Aging, as a primary risk factor for various acute and chronic NDDs, leads to changes in the expression of Hb that are closely related to various pathological processes [56]. Here, we summarize alterations in circulating and cerebral Hb in different species, including mice, monkeys, and humans, alongside associated pathological alterations (Figure 1). Previous studies have shown that Hb levels in the blood decrease significantly with age in humans [57], and this decline in circulating Hb is closely associated with mitochondrial dysfunction, disturbances in oxygen homeostasis, and cognitive decline [58]. In contrast, non-anemic elderly individuals with higher levels of circulating Hb exhibit better physical functionality. Moreover, this decline is positively correlated with disrupted iron metabolism [40,59].

The expression of cerebral Hb is also known to decrease with age. In aged mice, Hb levels in the brain have been shown to decrease with age, accompanied by changes in mitochondrial distribution [53]. In monkeys and humans, the level of neuronal Hb in the striatum also declines with age, although the underlying causes and functional implications remain unclear [60,61]. Collectively, these findings suggest that Hb could serve as a potential therapeutic target for age-related neurodegenerative diseases.

### 3.3. Regulation of Hb by Hypoxic Stress

It is well established that hypoxia contributes to the progression of various age-related NDDs. However, the precise role of hypoxic stress in such diseases remains unclear [11,12,13]. Hypoxic stress is closely associated with the expression of hypoxia-inducible factor (HIF) target genes, many of which play crucial roles in the adaptive response of the body to stress. For example, the HIF target genes involved in angiogenesis and glucose metabolism are known to be activated by the HIF pathway [9,62,63]. Hb responds to hypoxic stress through modulations in oxygen affinity and expression dynamics; these processes are intimately associated with the HIF pathway. For instance, in hypoxic environments, the red drum fish (*Sciaenops ocellatus*) adjusts its Hb expression levels and increases the affinity of Hb for O_2_ to improve respiratory performance under hypoxic conditions [64]. Acute and chronic hypoxia is known to induce a significant increase in Hb expression in the brains and skeletal muscles of mice [65]. Similarly, extreme hypoxia has been shown to induce the upregulation of Hb in the retinal ganglion cells of rats and human glioblastoma multiforme cells [66,67]. Humans who live at high altitudes exhibit adaptations by adjusting Hb levels and Hb-O_2_ affinity to mitigate the effects of arterial hypoxemia [68,69,70]. Collectively, these studies indicate that under hypoxic stress, the body regulates Hb by modifying its structure to adjust oxygen affinity and the expression levels of Hb. Notably, these changes occur not only in circulating Hb in erythroid cells but also in cerebral Hb and other non-erythroid tissues where Hb is expressed abnormally.

**Figure 2:**
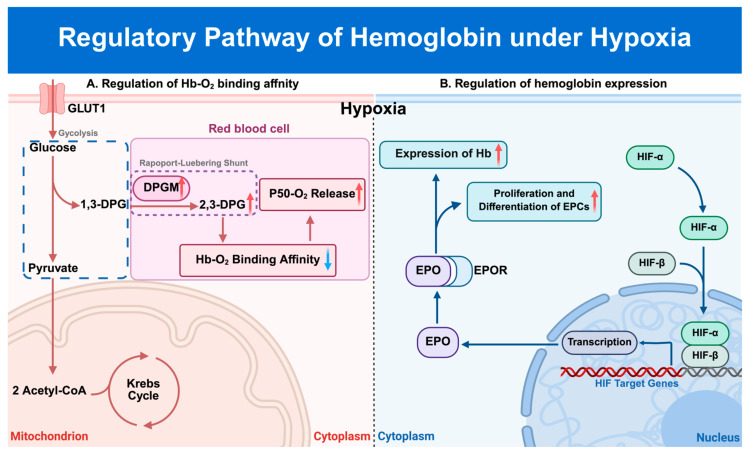
Regulation of hemoglobin levels under hypoxic stress. (**A**) Hypoxia promotes the increased activity of 2,3-DPG isomerase in red blood cells, inducing the production of the glycolytic byproduct 2,3-DPG. Elevated levels of 2,3-DPG adjust the affinity of hemoglobin for oxygen, thereby increasing the levels of P50 and enhancing oxygen release to peripheral hypoxic tissues. This mechanism helps to prevent hypoxia-induced tissue inflammation and damage. (**B**) Hypoxia promotes the nuclear translocation of HIF-α, which then binds with HIF-β to mediate the transcription of downstream genes such as *EPO*. The increased expression of EPO binds to its receptor, EPOR, subsequently inducing hemoglobin expression and promoting the proliferation and differentiation of erythroid precursor cells. 2,3-DPG: 2,3-diphosphoglycerate, DPGM: phosphoglycerate mutase, HIF-α: hypoxia-inducible factor subunit α, HIF-β: hypoxia-inducible factor subunit β, EPO: erythropoietin, EPOR: erythropoietin receptor, EPC: erythroid precursor cell. “↑”: hypoxia promotion pathway, “↓”: hypoxia inhibition pathway.

The precise molecular mechanisms by which the body adjusts Hb-O_2_ affinity under hypoxia remain contentious. Some researchers argue that hypoxia-induced changes in the metabolism of red blood cells lead to an increase in intracellular 2,3-diphosphoglycerate (2,3-DPG), an allosteric modulator that reduces Hb-O_2_ affinity. This reaction facilitates oxygen unloading to target tissues, thus reducing the difficulty of oxygen uptake under hypoxic conditions (Figure 2A) [71,72,73]. Conversely, many mammals living at high altitudes exhibit an increase in Hb-O_2_ affinity when compared to their lowland relatives, potentially as an adaptive mechanism to resist hypoxic damage [74,75,76,77,78]. Thus, the precise molecular mechanisms governing Hb-O2 affinity under hypoxia remain a topic of ongoing debate.

Hypoxia also regulates the expression of Hb, primarily via the HIF pathway. HIF-1, a transcription factor, binds to the hypoxia response element (HRE) in the 3′-flanking region of the human *EPO* gene, which encodes erythropoietin (EPO), the hormone that controls the production of red blood cells, and thus, the oxygen-carrying capacity of the blood [79,80,81]. Injecting or genetically overexpressing EPO in the brain of mice can upregulate neuronal Hb, thus enhancing mitochondrial activity, and helping the brain to resist hypoxia (Figure 2B) [47,54]. Collectively, these studies demonstrated a close regulatory relationship between hypoxic stress and Hb in various non-erythroid tissues and cells, though the exact regulatory pathways remain to be elucidated.

Moreover, existing studies suggest that the aberrant expression of both circulating and cerebral Hb may be involved in hypoxic-related NDDs [82,83]. However, there is currently insufficient evidence to fully describe the hypoxic response of Hb in the development of acute and chronic NDDs, or to interpret its precise function. Further investigations into this axis may yield critical insights into endogenous neuroprotective strategies.

## 4. Current and Potential Research on the Relationship Between Hb and Hypoxia-Related NDDs

Oxygen is essential for life, and hypoxia induces a cascade of pathological reactions that initiate or exacerbate many diseases [84]. It is well recognized that hypoxia is closely associated with the development of both acute and chronic NDDs [85,86,87]. Previous studies suggested that under hypoxic conditions, the body regulates Hb by modulating its oxygen affinity and upregulating Hb expression to augment oxygen delivery and exert ancillary, non-respiratory functions, thereby reducing the stress-induced damage caused by hypoxia [47,54,68,88]. Thus, the regulation of circulating and cerebral Hb may constitute a key adaptive mechanism in hypoxia-associated NDDs.

Based on this, we summarize the expression patterns and potential functions of Hb in various acute and chronic NDDs, emphasizing the non-oxygen-binding protective role of cerebral Hb in the brain. We also explore how alterations in circulating Hb correlate with disease states and their potential prognostic implications. Collectively, these findings position Hb as a multifaceted player in NDDs and a promising target for mechanistic and translational research.

### 4.1. Hb and Acute NDDs

#### 4.1.1. Stroke

**Figure 3:**
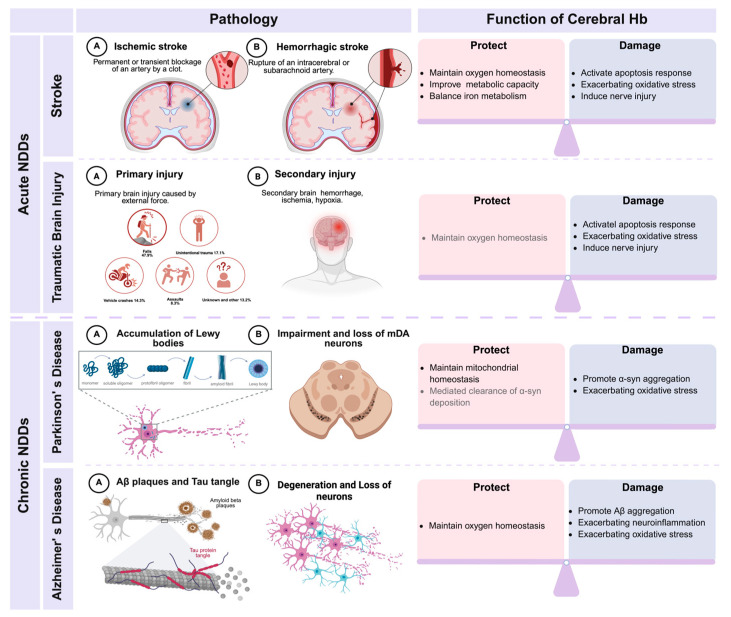
Cerebral hemoglobin in various hypoxia-related NDDs: current and potential mechanisms. The black font represents the functionality of hemoglobin confirmed by existing research, while the gray font represents the potential functionality of hemoglobin that has yet to be experimentally verified. Aβ: amyloid-beta, α-syn: alpha-synuclein, Hb: hemoglobin, mDA neurons: midbrain dopaminergic neurons, NDDs: neurodegenerative diseases.

Ischemic stroke (IS) is primarily caused by the interruption of cerebral blood flow, leading to severe neural damage; this represents one of the leading global causes of death and disability [36,89,90]. The blockage of cerebral blood flow results in oxygen and glucose deprivation, a crucial step in the pathophysiology of IS that directly disrupts ATP production, ionic homeostasis, and acid–base equilibrium [91,92]. While irreversible damage occurs in the infarct core, the surrounding penumbra and ischemic regions may resist further injury by increasing oxygen supply. Reperfusion or collateral circulation is critical in the restoration of oxygen supply and the prevention of pathological deterioration [93,94]. As the main oxygen-transporting protein, the cerebral Hb expression may influence penumbral viability [95,96]. Studies in animal models of IS have shown that the levels of neuronal Hb increase significantly after IS and that ischemic preconditioning can provide neuroprotection by enhancing the expression of neuronal Hb (Figure 3) [97,98]. These findings suggest that the compensatory upregulation of cerebral Hb may serve as an essential self-protective mechanism following IS. Furthermore, clinical studies have demonstrated that lower levels of circulating Hb are positively correlated with stroke severity and progression, indicating its critical role in the development of IS [99,100,101].

Unlike IS, hemorrhagic stroke (HS) results from the vascular rupture of blood vessels and the leakage of blood into brain tissue, thus leading to local damage. Common types of HS include intracerebral hemorrhage and subarachnoid hemorrhage [25]. In HS, extracellular free Hb is released from ruptured red blood cells; this can induce harmful oxidative stress, activate the caspase cascade, and lead to neuronal death, thus contributing to severe neurological damage [102,103,104,105,106]. Thus, extracellular Hb can exert toxic effects, while intracellular Hb may perform protective roles, although the specific mechanisms involved have yet to be fully elucidated.

#### 4.1.2. TBI

Traumatic brain injury (TBI) is a complex, multifaceted pathological process that involves both primary and secondary injury phases that contribute to progressive neurodegeneration [107,108]. In both stages, exogenous Hb that is released as a consequence of trauma plays a key role in the induction of neuronal damage, much mirroring mechanisms seen in HS [109]. This extracellular form of Hb not only exhibits cytotoxicity but also consumes NO and produces reactive oxygen species (ROS), which cause further neuronal necrosis [110,111].

However, Hb should not be viewed solely as a toxic agent in TBI. Several clinical studies have focused on increasing the circulating levels of Hb by administering EPO, which can stimulate erythropoiesis and enhance oxygen metabolism, thereby helping patients to resist injury. However, clinical results remain inconsistent, and the underlying molecular mechanisms require further investigation [112,113,114,115]. Therefore, characterizing the dual roles of Hb may inform novel therapeutic strategies for TBI.

### 4.2. Hb and Chronic NDDs

#### 4.2.1. Parkinson’s Disease

Parkinson’s disease (PD) is a chronic neurodegenerative disorder characterized by motor dysfunction, tremors, muscle rigidity, and postural instability; aging is considered to be the most significant risk factor [116,117,118]. The primary pathological features of PD include the progressive loss of dopaminergic neurons in the substantia nigra and the pathological accumulation of Lewy bodies [119]. Recent studies have shown that hypoxia is closely associated with the pathogenesis of PD and plays a critical role at multiple stages of the disease process [120,121]. As the key protein responsible for gas exchange, Hb is also involved in the pathogenesis of PD [122].

Studies relating to the cerebral expression of Hb in PD patients have found that the detection of Hb in post-mortem dopaminergic neurons implies a potential involvement in PD pathology (Figure 3) [20]. Dopaminergic neuronal loss is a significant hallmark of PD [123]. Gene expression analysis of dopaminergic cell lines overexpressing Hb has revealed that approximately 46% of genes encoding mitochondrial complexes were induced, indicating that Hb is an important regulator of mitochondrial function under both normal and pathological conditions [20]. Gene enrichment analysis further revealed that the overexpression of Hb led to significant alterations in key pathways related to oxygen homeostasis, oxidative phosphorylation, oxidative stress, and iron metabolism in dopaminergic neurons, all of which are closely related to the pathogenesis of PD, thus suggesting that Hb may play a protective role in the pathology of PD [124,125].

However, recent studies have also shown that the overexpression of Hb in the substantia nigra of mice can lead to Hb aggregation and motor learning deficits [46,126]. Furthermore, in dopaminergic cell lines, Hb overexpression induces transcriptional changes in genes involved in iron metabolism, potentially promoting iron dysregulation within the substantia nigra. This, in turn, may enhance free radical generation and oxidative stress, further impairing mitochondrial function in neurons [20]. While both studies investigated the consequences of Hb overexpression, their findings diverged in terms of the primary pathological outcomes—one emphasizing behavioral and protein aggregation phenotypes in vivo [46,126], and the other highlighting molecular and metabolic disruptions at the cellular level [20]. These differences may arise from variations in experimental models, cellular context, or the degree of Hb overexpression, underscoring the ongoing debate regarding the precise role of Hb in dopaminergic neuron function and vulnerability.

The misfolding and aggregation of alpha-synuclein (α-syn) leads to the pathological formation of Lewy bodies in the brain, a key hallmark of PD [127,128,129]. Previous studies showed that the cytoplasmic accumulation of α-syn increases the formation of Hb-α-syn complexes within cytosolic and mitochondrial components; however, whether this complex plays a role in the pathology of PD has yet to be investigated [61]. In brain tissues from PD patients, Hb has been detected in Lewy bodies associated with pathological α-syn deposits, although the mechanisms underlying the aggregation of neuronal Hb, α-syn accumulation, and Hb-α-syn complex formation remain unclear [130].

Furthermore, multiple case–control studies have indicated that anemia, triggered by reduced levels of circulating Hb, is an important risk factor for PD [131,132]. However, whether there is a causal relationship between reduced levels of circulating Hb and the progression of PD has yet to be specifically investigated. In summary, Hb may contribute to the development and progression of PD via multiple mechanisms, but its specific role and potential therapeutic applications require further clarification. Therefore, studies are now needed to investigate the relationship between the central levels of Hb and the progression of PD to elucidate the precise function of neuronal Hb in PD.

#### 4.2.2. Alzheimer’s Disease

Alzheimer’s disease (AD) is a chronic neurodegenerative disease that predominantly affects the elderly and is characterized by progressive cognitive impairment and behavioral changes. AD is the most common form of dementia [133,134] and is closely associated with microvascular dysfunction in the brain, as well as defects in the blood–brain barrier, both of which reduce cerebral perfusion and thus oxygen and nutrient supply to the brain. This implies that hypoxia plays a significant role in the pathogenesis of AD [135,136]. The key pathological features of AD include the accumulation of amyloid β-protein (Aβ) plaques and the hyperphosphorylation and aggregation of tau protein [137]. Hb has also been implicated in the development and progression of AD, although its exact role has yet to be identified [138,139].

By reviewing the expression of Hb in AD patients and animal models, it was evident that Hb is widely expressed in neurons and glial cells in the brain [82,140,141], though its exact function remains controversial (Figure 3). Some researchers suggest that this aberrantly expressed Hb binds to Aβ, thus promoting its abnormal deposition and exacerbating neuroinflammation and neuronal injury. Subsequently, this process can lead to mitochondrial dysfunction, oxidative stress, neuronal apoptosis, and synaptic disruption [140,141,142]. On the other hand, several clinical cohort studies have indicated that lower circulating levels of Hb are associated with a higher risk of AD, thus suggesting that Hb may also exert endogenous protective effects during the progression of AD [143,144].

In summary, research on the precise functionality of Hb in AD is still in its infancy and requires further investigation of the relationship between cerebral Hb levels and AD progression. This highlights the need for future studies to investigate Hb as a potential target for the prevention and treatment of AD.

#### 4.2.3. Amyotrophic Lateral Sclerosis

Amyotrophic lateral sclerosis (ALS) is a devastating motor neuron disease that typically leads to paralysis and death due to respiratory failure [145]. Defective hypoxia signaling is thought to be a significant factor contributing to the degeneration of motor neurons in ALS [146]. While clinical studies have demonstrated an association between lower circulating levels of Hb and ALS mortality, this relationship was not statistically significant [147]. Nonetheless, given the critical role of hypoxia in the pathogenesis of ALS and the regulation of Hb under hypoxic conditions, the further investigation of cerebral and circulating Hb in ALS may yield mechanistic insights into ALS pathophysiology and contribute to the development of effective therapies.

## 5. Discussion and Future Directions

Whether in IS, HS, TBI, or chronic neurodegenerative diseases, such as PD, AD, and ALS, aging constitutes a principal driver of neuropathological initiation and progression. Given the strong association between hypoxia and NDDs, it is essential to investigate the potential functions and mechanisms of Hb in these conditions [9]. Existing research primarily focused on changes in the circulating levels of Hb and their relationship to disease progression, exploring whether the regulation of Hb expression could influence disease outcomes. Notably, Hb appears to exert both neuroprotective and neurotoxic effects under different pathological contexts—a duality that raises the key question: what determines the shift between its beneficial and detrimental roles?

Beyond its systemic function, hemoglobin expressed within the brain—particularly in neurons—has been implicated in the regulation of key pathophysiological processes in neurodegenerative diseases. In IS models, cerebral Hb contributes to the preservation of oxygen and iron homeostasis in peri-infarct regions, suggesting a compensatory, neuroprotective response to acute hypoxic injury [96,97]. In PD models, elevated neuronal Hb has been associated with mitochondrial function, oxidative stress regulation, and iron metabolism, implicating it in broader cellular stress responses [124,125]. These observations underscore the context-dependent nature of cerebral Hb activity across disease states and reinforce the need to delineate the conditions under which it confers protection versus those in which it contributes to pathology.

Despite its protective potential, cerebral hemoglobin can also contribute to neurodegenerative pathology. In AD models, Hb has been reported to interact with amyloid-β, facilitating its aggregation and deposition [140]. Similarly, in PD models, Hb co-localizes with α-synuclein within Lewy bodies, potentially exacerbating mitochondrial dysfunction and neuronal damage [130]. These findings suggest that cerebral Hb may function as a molecular nexus linking physiological aging with pathological processes, with its effects shaped by specific protein interactions and cellular environments. Expanding this framework, it is pertinent to explore whether Hb similarly engages with other disease-related proteins, such as TDP-43 in ALS, to elucidate its broader role across neurodegenerative disorders.

The divergent roles of cerebral Hb—ranging from neuroprotective to neurotoxic—likely reflect a complex interplay of multiple factors. One major determinant is its expression level: modest increases in neuronal Hb may enhance oxygen buffering and mitochondrial function [148], whereas excessive accumulation could disrupt redox homeostasis or promote pathological protein interactions [122]. The type of cell expressing Hb is another key consideration. While neuronal Hb has been associated with protective functions [20], its presence in activated glial cells—particularly microglia—may amplify inflammatory responses or oxidative stress under disease conditions [149]. Moreover, the local microenvironment, including metabolic stress, redox status, and the presence of pathogenic protein aggregates, can shape the functional consequences of Hb expression. These context-dependent effects may help explain the conflicting observations across different neurodegenerative disease models and highlight the need for fine-resolution studies that integrate spatial, temporal, and cellular dimensions of Hb dynamics.

In non-neurological conditions such as chronic kidney disease anemia, β-thalassemia, and sickle cell disease, therapeutic strategies aimed at modulating hemoglobin expression—most notably through EPO supplementation or HIF pathway modulation—have been extensively studied and clinically validated [150,151,152]. These interventions have demonstrated efficacy in restoring systemic oxygen transport and improving patient outcomes. However, when applied to central nervous system disorders, the therapeutic value of EPO-mediated hemoglobin regulation remains contentious. Some studies suggest neuroprotective effects of EPO in acute injury models, including IS and TBI, potentially through the upregulation of cerebral Hb and anti-apoptotic signaling [47,153,154]. Conversely, other investigations report limited or even adverse outcomes, raising concerns about the timing, dosage, and target specificity of EPO-based therapies in neurodegenerative contexts [154]. These inconsistencies underscore the need for more nuanced, disease-specific assessments of Hb-targeted interventions in NDDs, with particular attention to the cellular context, stage of disease progression, and systemic versus cerebral effects.

Cerebral Hb may also bridge the gap between acute and chronic NDDs. In animal models of IS, the levels of Hb increase following cerebral ischemia [10,97], and hypoxia induces the upregulation of cerebral Hb; however, this phenomenon occurs predominantly in neurons [155]. In the short term, the increased expression of cerebral Hb appears to be a compensatory mechanism that helps the brain resist both ischemic and hypoxic damage. However, as more research focuses on post-stroke cognitive impairment and the commonalities between stroke and PD, it has become critical to investigate the potential link between acute and chronic NDDs from the perspective of Hb [156,157,158,159]. These observations raise the possibility that sustained or dysregulated Hb expression following stroke may contribute to the pathophysiological continuum from acute injury to chronic neurodegeneration. Whether this represents a causal mechanism or a secondary consequence remains unresolved and warrants systematic investigation. Elucidating the temporal trajectory and functional outcomes of Hb elevation after acute brain injury may offer novel insights into shared molecular pathways across NDDs and inform strategies for early intervention.

Cerebral Hb exhibits considerable heterogeneity in its expression patterns across different NDDs, both in terms of overall levels and cellular localization (Table 2). Although cerebral Hb has been primarily detected in neurons in both normal aging and NDDs, its expression patterns are inconsistent. Hb levels gradually decrease during normal aging, while in chronic NDDs associated with abnormal aging, cerebral Hb levels tend to increase. Although hypoxia is defined as a common pathological feature of both aging and NDDs, attributing changes in cerebral Hb expression solely to hypoxia regulation may be overly simplistic. We propose that regulatory pathways beyond HIF may be involved in these pathological conditions. Investigating these pathways could offer new insights into the mechanisms underlying NDDs and provide potential intervention strategies for other Hb-related diseases such as thalassemia and sickle cell anemia.

In addition to Hb, neuroglobin (Ngb) has also been implicated in neuroprotection across various neurological conditions. Its expression is often negatively correlated with disease progression and functional decline, particularly in acute settings such as stroke [160,161,162]. While Ngb has been reported to translocate to mitochondria under hypoxic stress—potentially enhancing oxygen utilization—findings remain inconsistent regarding whether hypoxia reliably induces Ngb expression in neurons or brain tissue [155,163,164]. Despite these discrepancies, the possibility that Ngb acts as a broader adaptive mechanism in hypoxic–ischemic injury warrants further exploration. Understanding its regulatory dynamics may provide additional insight into how oxygen-binding globins contribute to the pathophysiology of NDDs.

**Table 2:** Cerebral hemoglobin expression in hypoxia and related brain diseases. “+”: Positive cell markers in immunofluorescence, “↑”: upregulated level of Hbα/Hbβ, “↓”: downregulated level of Hbα/Hbβ, “-”: unchanged level of Hbα/Hbβ. BCAO: bilateral common carotid artery occlusion, dMCAO: distal middle cerebral artery occlusion, Hbα: hemoglobin alpha subunit, Hbβ: hemoglobin beta subunit, OGD: oxygen–glucose deprivation, UPDRS: Unified Parkinson’s Disease Rating Scale.

Stress/Disease	Species	Age/Disease Model/Disease Stage	Cell Type	Hb Expression	Functional Implication	Reference
Aging	Mouse	6/12/18 months	/	Hbα ↓, Hbβ ↓	Protective	[10]
	Cynomolgus	3–4/10–12/15 years	Neuron (Non-marker)	Hbα ↓, Hbβ ↓	Protective	[61]
Hypoxia	Mouse	7% O_2_ treatment for 28 days	Neuron (Map2+)	Hbα ↑	Protective	[155]
Stroke	Rat	dMCAO	Neuron (NeuN+)	Hbα ↑, Hbβ ↑	Protective	[97]
	Rat	OGD	Neuron (Primary cell)	Hbα ↑, Hbβ ↑	Protective	[97]
	Mouse	BCAO	Neuron (Non-marker)	Hbα ↑	Protective	[10]
Parkinson’s disease	Patient	Braak stage 0, I, II	Neuron (Non-marker)	Hbα -, Hbβ -	Unclear	[82]
	Patient	Braak stage III, IV, V, VI	/	Hbα -, Hbβ -	Unclear	[165]
	Patient	UPDRS = 54, 70, 45.5	Neuron (Non-marker)	Hbα -, Hbβ -	Unclear	[130]
Alzheimer’s disease	Patient	Braak stage III	Neuron (Non-marker)	Hbα -, Hbβ -	Unclear	[82]
	Patient	Braak stage V, VI	Neuron (Non-marker)	Hbα -, Hbβ -	Pathogenic	[140]
	Mouse	APP/PS1transgenic	Neuron (NeuN+) Oligodendrocyte (OSP+) Astrocyte (GFAP+) Microglia (Iba1+)	Hbα ↑	Pathogenic	[141]

## 6. Conclusions

In conclusion, this review consolidates current knowledge on the multifaceted roles of hemoglobin in neurodegenerative diseases, highlighting its significant contribution to disease pathogenesis. Future investigations should be systematically structured along three principal directions. Firstly, comprehensive genetic analyses are warranted to identify hemoglobin-related variants and mutations that may influence disease susceptibility and progression, a domain that remains insufficiently characterized. Secondly, in-depth cellular and molecular studies are necessary to delineate the cell type-specific functions of hemoglobin within the central nervous system and to elucidate the regulatory mechanisms governing its expression and activity. Thirdly, translational research should prioritize the development and evaluation of targeted interventions aimed at modulating hemoglobin expression or function. Experimental strategies, including conditional knockout models and the modulation of hypoxia-inducible factor signaling pathways, may provide valuable tools for these endeavors. Such integrated approaches are expected to advance mechanistic insights and facilitate the identification of novel biomarkers and therapeutic targets, thereby contributing to improved management of neurodegenerative disorders.

## Data Availability

Not applicable.

## Abbreviations

The following abbreviations are used in this manuscript:

2,3-DPG	2,3-diphosphoglycerate
Aβ	Amyloid β-protein
AD	Alzheimer’s disease
ALS	Amyotrophic lateral sclerosis
α-syn	Alpha-synuclein
CNS	Central nervous system
DPGM	Phosphoglycerate mutase
EPO	Erythropoietin
EPOR	Erythropoietin receptor
EPC	Erythroid precursor cell
Hb	Hemoglobin
Hbα/β	Hemoglobin alpha/beta subunit
HIF	Hypoxia-inducible factor
HIF-α/β	Hypoxia-inducible factor subunit α/β
HRE	Hypoxia response element
HS	Hemorrhagic stroke
H_2_O_2_	Hydrogen peroxide
IS	Ischemic stroke
mDA neurons	Midbrain dopaminergic neurons
NDDs	Neurodegenerative diseases
Ngb	Neuroglobin
NO	Nitric oxide
PD	Parkinson’s disease
ROS	Reactive oxygen species
TBI	Traumatic brain injury
